# EFIMD-Net: Enhanced Feature Interaction and Multi-Domain Fusion Deep Forgery Detection Network

**DOI:** 10.3390/jimaging11090312

**Published:** 2025-09-12

**Authors:** Hao Cheng, Weiye Pang, Kun Li, Yongzhuang Wei, Yuhang Song, Ji Chen

**Affiliations:** 1 School of Computer and Information Security, Guilin University of Electronic Technology, Guilin 541004, China; chenghao@guet.edu.cn (H.C.); 395708917@mails.guet.edu.cn (W.P.); songyuhang@mails.guet.edu.cn (Y.S.); 2 School of Information Engineering, Guilin Institute of Information Technology, Guilin 541100, China; 22032303054@mails.guet.edu.cn; 3 School of Mathematical and Computational Sciences, Guilin University of Electronic Technology, Guilin 541004, China; chenji@guet.edu.cn

**Keywords:** deep forgery detection, feature interaction, multi-domain fusion, EFIMD-Net, spatial-frequency features

## Abstract

Currently, deepfake detection has garnered widespread attention as a key defense mechanism against the misuse of deepfake technology. However, existing deepfake detection networks still face challenges such as insufficient robustness, limited generalization capabilities, and a single feature extraction domain (e.g., using only spatial domain features) when confronted with evolving algorithms or diverse datasets, which severely limits their application capabilities. To address these issues, this study proposes a deepfake detection network named EFIMD-Net, which enhances performance by strengthening feature interaction and integrating spatial and frequency domain features. The proposed network integrates a Cross-feature Interaction Enhancement module (CFIE) based on cosine similarity, which achieves adaptive interaction between spatial domain features (RGB stream) and frequency domain features (SRM, Spatial Rich Model stream) through a channel attention mechanism, effectively fusing macro-semantic information with high-frequency artifact information. Additionally, an Enhanced Multi-scale Feature Fusion (EMFF) module is proposed, which effectively integrates multi-scale feature information from various layers of the network through adaptive feature enhancement and reorganization techniques. Experimental results show that compared to the baseline network Xception, EFIMD-Net achieves comparable or even better Area Under the Curve (AUC) on multiple datasets. Ablation experiments also validate the effectiveness of the proposed modules. Furthermore, compared to the baseline traditional two-stream network Locate and Verify, EFIMD-Net significantly improves forgery detection performance, with a 9-percentage-point increase in Area Under the Curve on the CelebDF-v1 dataset and a 7-percentage-point increase on the CelebDF-v2 dataset. These results fully demonstrate the effectiveness and generalization of EFIMD-Net in forgery detection. Potential limitations regarding real-time processing efficiency are acknowledged.

## 1. Introduction

With the rapid development of generative artificial intelligence technology, the generation of deepfake content has become more convenient and realistic. This kind of technology can easily replace the face in the video, tamper with the expression and movement of the character, and even synthesize the complete audio and video clips, and its high fidelity makes it difficult for the naked eye to distinguish between forgery and authenticity. Although deep forgery technology has shown positive application prospects in the fields of film production, virtual reality, education and entertainment, its abuse has also led to many serious social problems, such as malicious fabrication and dissemination of false information, invasion of personal privacy, financial fraud, etc., posing a serious threat to social order and network security [1,2]. Deep forgery detection, as a key defense against the abuse of such technologies, has attracted wide attention from academia and industry in recent years, and has made remarkable progress. The existing detection methods mainly have the following three core challenges when dealing with increasingly complex and diverse forgery attacks:

First, most methods rely on single modal or single domain features (such as only spatial domain or only frequency domain), and it is difficult to fully capture the complex traces left by counterfeit content in multiple dimensions. For example, spatial domain methods may ignore weak but critical counterfeit fingerprints in the frequency domain, while frequency domain methods may lose important spatial structure and semantic information [3,4]. Specifically, methods relying primarily on spatial or frequency domain information have major limitations. The spatial domain excels at capturing obvious visual flaws, but fails to detect high-frequency artifacts—such as those from GANs [5] and diffusion models [6]—in the frequency domain. Moreover, when forgery quality is extremely high (leading to weak spatial clues), over-reliance on spatial features causes overfitting. This results in poor generalization and robustness when facing unknown forgery techniques or cross-dataset scenarios. Although the frequency domain is good at capturing significant periodic noise and abnormal specific frequency components, excessive focusing on frequency domain information will lead to the model ignoring image spatial structure and advanced semantic content. Spatial clues such as object logical relationship and facial organ arrangement are also critical to forgery detection. Pure frequency domain analysis cannot effectively utilize such information. This results in limited detection accuracy in complex scenes and ability to distinguish forgery types such as semantic inconsistency splicing. Recent work by Miao et al. [7] has demonstrated the effectiveness of high-frequency fine-grained features in face forgery detection through their F^2^Trans framework, yet their approach still primarily focuses on frequency domain analysis without fully addressing the spatial–frequency domain integration challenge.

Secondly, some methods that attempt to fuse multi-domain features only use simple fusion strategies (such as concatenation or element-wise addition), which cannot fully utilize the effective interaction and complementary information enhancement between different features, thereby limiting the model’s deep understanding of forgery patterns [8]. Advanced frameworks like UMMAFormer by Li et al. [9] employ multimodal adapters for temporal forgery localization, demonstrating improved feature interaction across domains, but they often require complex training paradigms that may not generalize well to unseen forgery types. How to effectively fuse multi-domain features remains an unsolved problem.

Third, existing detection models are typically optimized for specific forgery algorithms in known datasets, leading to overfitting to forgery traces in the training data. When faced with new forgery techniques or data with different distributions, performance drops sharply, and generalization ability is weak [10,11]. This indicates that generalization ability across datasets and forgery types, especially adaptability to complex and covert new forgery attacks, still needs to be further improved.

To solve the above problems, this paper proposes a deepfake detection network called EFIMD-Net, which is based on enhanced feature interaction and multi-domain fusion. The dual-stream parallel architecture is used to process RGB information and noise residual features extracted by SRM filter bank, respectively, and three core modules are designed to realize efficient interaction and depth fusion of spatial and frequency domain features, so as to improve the accuracy and generalization of detection.

Domain refers to the distribution in which the input features are located. In this paper, domain refers to the spatial domain (RGB stream) and the frequency domain (SRM stream). In order to solve the problem that spatial and frequency domain features are not complementary enough, we designed a Cross-feature Interaction Enhancement (CFIE) module, which dynamically guides the interaction of two domain features through cosine similarity instead of simple static fusion. In order to solve the problem that existing models are not able to detect subtle and diverse forgery traces and multi-domain feature guidance is not clear, we introduce Enhanced Feature Guidance (EFG) module, which integrates multi-dimensional attention mechanism to enable the network to extract semantic information from RGB streams more accurately to guide SRM streams to focus on potential forgery regions. Finally, we developed an Enhanced Multi-scale Feature Fusion (EMFF) module, which efficiently integrates features at different network levels through adaptive feature enhancement and recombination techniques. The three core modules are designed to more comprehensively extract and utilize complementary information in the spatial domain and frequency domain, thereby significantly improving the model’s detection accuracy and generalization capabilities for various types of deepfake content.

The main contributions of this study can be summarized as follows:

1. This paper proposes a cosine similarity-guided Cross-feature Interaction Enhancement (CFIE) mechanism, which uses dynamic cosine similarity calculation to guide space–frequency domain feature interaction instead of traditional static fusion mode to achieve efficient adaptive fusion between features and effectively improve the complementarity of multi-domain features.
2. An Enhanced Feature Guidance (EFG) module integrating multi-level self-attention, channel attention [12] and spatial attention is designed to extract semantic information accurately from RGB streams through multi-dimensional attention mechanism, guide SRM streams to locate potential forgery areas, and enhance the ability to capture and locate diverse fine artifacts.
3. An Enhanced Multi-scale Feature Fusion (EMFF) technique is developed, which effectively improves the perception, integration, and discrimination ability of the model for different scale forgery artifacts through adaptive feature enhancement and efficient recombination strategy.

Finally, extensive experiments conducted on multiple standard datasets demonstrate that EFIMD-Net exhibits higher effectiveness and robustness in deepfake detection tasks, achieving superior fake detection results.

## 2. Materials and Methods

### 2.1. Related Work

#### 2.1.1. Deep Forgery Technology

Early deep forgery techniques relied primarily on autoencoders to achieve face replacement, as in December 2017 DeepFakes [13] reconstructed facial features of one person onto another’s face by training shared encoders and independent decoders between source and target faces. Subsequently, methods based on computer graphics and more complex deep learning models, such as Face2Face [14], further improved the visual and realistic feel of face replacement by capturing the target person’s expression in real time to drive the source person’s facial animation, and FaceSwap [15]. In recent years, the development of generative adversarial networks (GANs) has greatly advanced deep forgery techniques, such as StyleGAN [16] and its successor StyleGAN2 [17], which can generate high-resolution images that are visually indistinguishable from real faces. More recently, techniques based on diffusion models, such as Stable Diffusion [18] and DALL-E 2 [19], have not only excelled at face synthesis, but also extended the scope of fake content to a wider range of images and video scenes, reaching new heights of quality and diversity.

#### 2.1.2. Multi-Domain Feature Fusion Detection Method

Spatial feature-based detection methods mainly focus on visual artifacts, semantic inconsistencies and abnormal physiological features introduced by forgery process in images or video frames. Earlier studies, such as the work of Li et al. [20], attempted to detect fake videos by analyzing the dynamic unnaturalness of facial keypoints and expressions, while Yang et al. [21] in 2019 focused on analyzing the dissonance between head poses and movements of other body parts. In recent years, Rossler et al. [22], in their landmark work, systematically compared the performance of various CNN architectures on FaceForensics++ (FF++) datasets and found that Xception networks [23] showed superior performance on this task. Follow-up studies, such as the Multi-Attention Deepfake Detection (MAT) method proposed by Zhao et al. [24], try to integrate and weight spatial features extracted from different network levels by introducing attention mechanism so as to improve the detection effect. These detection methods combined with a CNN gradually become mainstream.

Frequency-domain feature-based analysis methods focus on image features after frequency transformation (such as Fourier transform, discrete cosine transform, etc.). An early study by Durall et al. [25] in 2020 found systematic differences in the statistical properties of the spectrum between real and fake images, e.g., fake images may introduce periodic artifacts in the spectrum due to upsampling operations. Frank et al. [26] further analyzed the spectral features of images generated by different GAN models using discrete cosine transform (DCT) to reveal the possible frequency fingerprints left by specific generation networks. Inspired by this, Li et al. [27] proposed a frequency-aware deep forgery detection network (FreqNet), which enhances the perception and generalization ability of the model for forgery traces by learning in frequency space. Tan et al. [28] designed a more complex multi-context and multi-frequency aggregation network (MkfaNet), which aims to adaptively process and fuse information from different frequency components.

Recent research trends tend to combine spatial and frequency domain (or even other domains such as noise domain) features in order to achieve more comprehensive and robust forgery detection capabilities. For example, the F3-Net proposed by Qian et al. [29] in 2020 considers both frequency inconsistency and statistical features in the frequency domain of images. Zhou et al. [30] designed a dual-stream network architecture to process RGB images (spatial domain) and noise features extracted by specific filters (which can be considered as a special kind of frequency-domain or residual-domain information). Qiu et al. [31] proposed D^2^Fusion (Dual-domain Fusion) framework, which enhances detection performance through specific dual-domain feature superposition and interaction strategies.

### 2.2. EFIMD-Net

#### 2.2.1. Overall Structure

**Figure 1:**
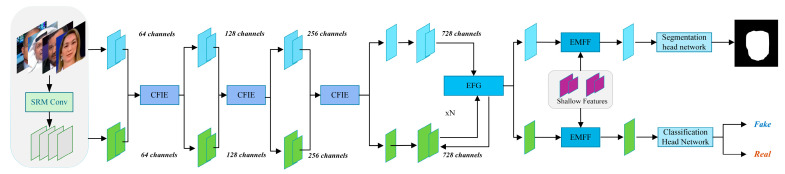
Overall architecture of enhanced feature interaction and multi-domain fusion deep forgery detection network.

The enhanced feature interaction and multi-domain fusion deep forgery detection network EFIMD-Net proposed in this paper adopts a dual-stream parallel architecture with Xception [23] as the backbone network. The upper branch of the network processes raw RGB images to capture macro semantic and visual features (RGB streams), and the lower branch processes noise feature maps extracted via SRM filter banks to focus on micro high-frequency artifacts and tamper traces (SRM streams). Firstly, RGB stream features and SRM stream features are fused through the Cross-feature Interaction Enhancement (CFIE) module, then RGB stream features are guided by the Enhanced Feature Guidance (EFG) module to make the model pay more attention to SRM features in key regions of the face, and then the Enhanced Multi-scale Feature Fusion (EMFF) module is responsible for integrating feature information from different depths and different processing stages of the network. The integration of multi-scale information enables the network to better perceive and locate fake artifacts with different performance on different scales. Finally, the network feeds the fused features into two independent prediction heads: a classification head and a segmentation head. The classification head predicts the overall authenticity of the input image through global information aggregation. The segmentation head outputs a pixel-level forgery region probability map to accurately locate specific tampering regions in the image. The overall architecture of the network is shown in Figure 1.

#### 2.2.2. Cross-Feature Interaction Enhancement Module

**Figure 2:**
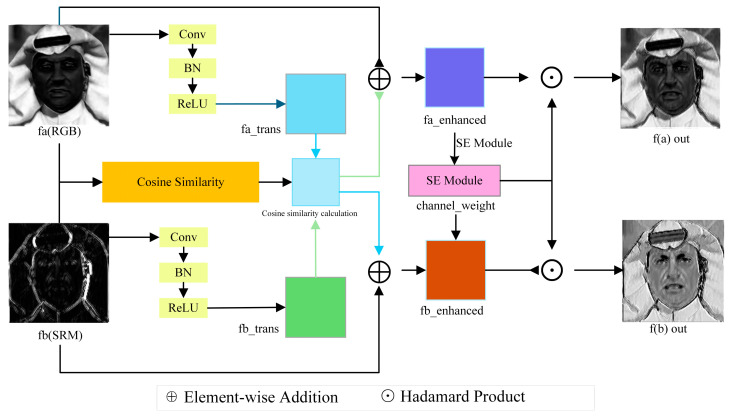
CFIE module structure.

The traces of deep forgery are often reflected in both the visual content (spatial domain) and subtle noise patterns (frequency domain) of the image. SRM filter can effectively extract high-frequency noise features from images, which contain a lot of detail information, especially subtle clues left over from the process of deep forgery, which are difficult for human eyes to detect. However, relying solely on SRM features may ignore the overall semantic information of the image, while relying solely on RGB features may fail to capture these high-frequency fake fingerprints. In order to solve the problem that spatial domain (RGB stream) and frequency domain (SRM stream) features are not fully utilized and effectively complemented, we design a Cross-feature Interaction Enhancement (CFIE) module, as shown in Figure 2. This module uses an adaptive interaction mechanism to control the interaction intensity of different domain features by using local similarity between features as a guiding signal, and it filters and strengthens the interactive features combined with a channel attention [12] mechanism. Finally, it integrates information through learnable residual connection to generate feature representation that is more sensitive to forgery traces and richer in information.

The core idea of CFIE module is to guide feature interaction through cosine similarity and enhance feature expression by using the channel attention mechanism. Given RGB stream characteristics fa and SRM stream characteristics fb, the CFIE module processing flow includes the following steps:

Cosine similarity between two feature streams is calculated as a guide signal for feature interaction.

(1) $$\cos\left(f_{a},f_{b}\right)=\frac{\sum_{c=1}^{C}f_{a}^{c}\times f_{b}^{c}}{\sqrt{\sum_{c=1}^{C}{\left(f_{a}^{c}\right)}^{2}}\times \sqrt{\sum_{c=1}^{C}{\left(f_{b}^{c}\right)}^{2}}}$$

where $f_{a}^{c}$ and $f_{b}^{c}$ denote the cth channel of features fa and fb, respectively, and C is the number of channels, this calculation is performed independently at each spatial position (h, w), and b is calculated for all batches of samples. Finally, cosine similarity graph with shape [B, 1, H, W] is obtained.

Then, feature transformation is applied to the two feature streams, respectively, to enhance the ability of feature expression, and cosine similarity is used to guide feature interaction to enhance complementary information. This design allows regions with high similarity to have stronger feature interactions, while regions with low similarity retain their own characteristics. Next, the channel attention mechanism is applied to further highlight important feature channels.

(2) $$C\left(x\right)=Sigmoid(Conv2d(\mathrm{ReLU}(Conv2d\left(\mathrm{AvgPool}2d\left(x\right)\right))))$$

where AvgPool2d is the global average pooling layer. Conv2d represents two-dimensional convolutions, respectively, and the weights are finally normalized by Sigmoid. Finally, residual connections are added to maintain the information flow, and the influence degree of interactive features is controlled by residual connections and learnable parameters.

(3) $$\mathrm{out}\text{\_}a=\gamma \;\times \;f_{a\_enhanced}\times C\left(f_{a\_enhanced}\right)+f_{a}$$

(4) $$\mathrm{out}\text{\_}b=\gamma \;\times \;f_{b\_enhanced}\times C\left(f_{b_{enhanced}}\right)+f_{b}$$

where γ is a learnable parameter initialized to 0 and gradually increasing with the training process.

#### 2.2.3. Enhanced Feature Guidance Module

**Figure 3:**
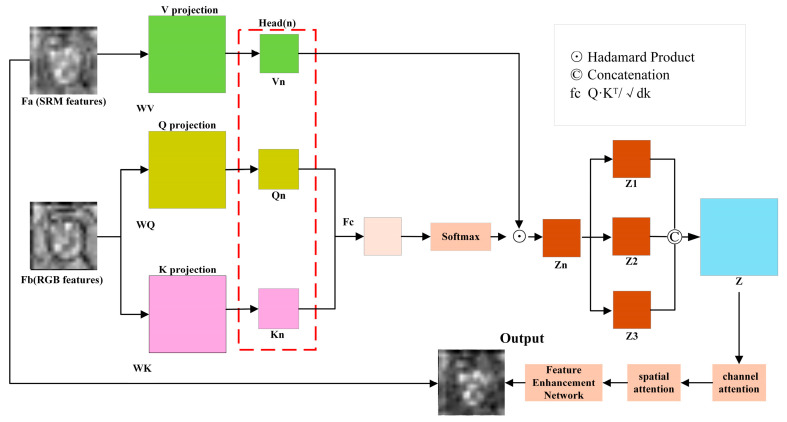
EFG module structure.

RGB feature stream can capture content information, semantic features, and advanced visual representation, and SRM feature stream can focus on capturing noise residuals, frequency-domain anomalies, and forged traces. In order to effectively fuse these two complementary information, create a more comprehensive feature representation, and further enhance the feature expression capability of the network, we design an Enhanced Feature Guidance (EFG) module, as shown in Figure 3. This module combines multi-attention, channel attention, and spatial attention mechanisms, uses RGB features to reveal the key areas of the face model, and guides the model to analyze and enhance SRM features in these key areas, so as to expose the forgery traces. This enhances the model’s ability to detect minute forgery traces.

The EFG module processes deep features from SRM streams $f_{a}$ and RGB streams $f_{b}$ as follows:

(5) $$\mathrm{Output}=\gamma \text{×}\mathrm{Enhance}(S(C(A\left(f_{a},f_{b}\right))))+\beta \times f_{a}$$

where A (∙) represents the multi-head self-attention, C (∙) represents the channel attention, and S (∙) represents the spatial attention.

First compute multi-head self-attention A (∙): divide the feature into three heads and compute the query, key, and value separately.

(6) $$Q\left(f_{b}\right)=Conv2d\left(f_{b},in\_channel,1\right)$$

(7) $$K\left(f_{b}\right)=Conv2d\left(f_{b},in\_channel,1\right)$$

(8) $$V\left(f_{a}\right)=Conv2d\left(f_{a},in\_channel,1\right)$$

Calculation of Attention Weights and Feature Reconstruction:

(9) $$A(f_{a},f_{b})=\left(\mathrm{Softmax}\left(Q\left(f_{b}\right)K{\left(f_{b}\right)}^{T}/{\sqrt{d}}_{k}\right)\right)V(f_{a})$$

where $f_{a}$ and $f_{b}$ are the characteristics of RGB stream and SRM stream, respectively, and d_k_ is the dimension of attention head. The multi-head attention mechanism enables multi-angle decomposition and reorganization of features. Each attention head can focus on feature expressions at different semantic levels, significantly improving the model’s ability to perceive complex forgery traces. Furthermore, the multi-head design allows the module to process feature information from different subspaces in parallel, enhancing the diversity and completeness of feature expressions while improving the module’s computational efficiency.

Channel attention C (∙) is then applied to the output of multi-focus attention to enhance important feature channels. Channel attention captures channel dependencies more accurately through adaptive averaging pooling and layer normalization.

(10) $$C\left(x\right)=Sigmoid(\mathrm{Conv}2d(\mathrm{ReLU}(\mathrm{LayerNorm}(\mathrm{Conv}2d(\mathrm{AvgPool}2d\left(x\right))))))\cdot x$$

Spatial attention S (∙) is then used, which calculates spatial attention through average pooling and maximum pooling to enhance the feature expression of key regions. Spatial attention can highlight the feature expression of counterfeit regions in images.

(11) $$S\left(x\right)=Sigmoid(\mathrm{Conv}2d(\mathrm{Concat}(\left(\mathrm{avg}\text{\_}\mathrm{out},\max\text{\_}\mathrm{out}\right))))x$$

where the global average pool and maximum pool on space ${avg}_{out}=AvgPool2d\left(x\right),\;\max_{out}=MaxPool2d\left(x\right)$

And then we conduct feature enhancement Enhance (∙):

(12) $$\mathrm{Enhance}\left(x\right)=Conv2d(\mathrm{ReLU}(\mathrm{DepthwiseConv}2d(\mathrm{Conv}2d\left(x\right))))$$

In this paper, we use depthwise separable convolution (DepthwiseConv2d) combined with ordinary convolution for feature enhancement, where DepthwiseConv2d reduces computational complexity while maintaining feature expressiveness.

Finally, residual connection and learnable parameters γ,β are used to control the degree of influence of enhanced features (reference Equation (5)). Residual connection guarantees the preservation of original feature information and avoids the loss of effective features.

#### 2.2.4. Enhanced Multi-Scale Feature Fusion Module

**Figure 4:**
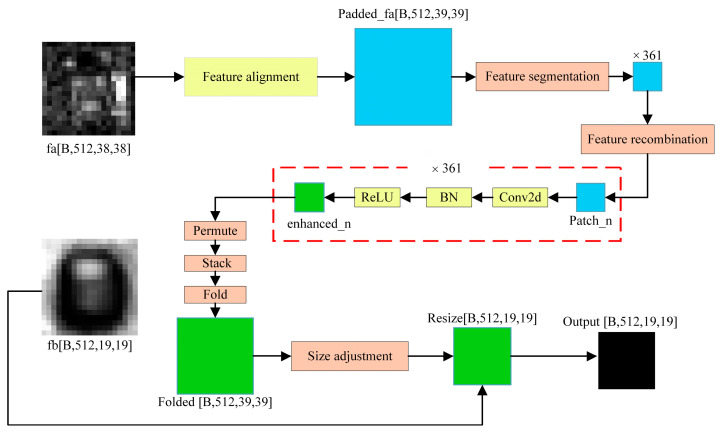
EMFF module structure diagram.

Since deep forgery may exhibit different features at different scales, multi-scale feature fusion is crucial. EMFF can effectively integrate feature information of different scales, transfer semantic information of high-level features to low-level features, while maintaining spatial details, creating a multi-scale feature representation with rich context, and enhancing the ability of the model to capture multi-scale forged traces. This module adopts feature recombination and enhancement strategy to realize deep interaction among features with different spatial resolutions and improve the discrimination ability and positioning accuracy of the model, as shown in Figure 4.

The EMFF module receives feature maps at two different scales, high-level features at a smaller scale $f_{b}$ and low-level features at a larger scale $f_{a}$. The specific processing flow is as follows:

(13) $$O=\gamma \times F(ReLU(E(R(U(Pad(f_{a})))))+\beta \times f_{b},\;Size(f_{a})\neq Size(f_{a})$$

$$\gamma \times F(ReLU(E(R(U(f_{a})))))+\beta \times f_{b},\;otherwise$$

where Pad represents padding, and if the size of the feature map does not match, use the padding operation to adjust the size of the input feature map $f_{a}$ so that it aligns with $f_{b}$. U (∙) means to expand the feature map $f_{a}$ into patches for subsequent processing. R (∙) denotes rearranging the unwrapped patches to fit the input format of the enhancement network E (∙). E (∙) denotes the feature-enhanced network, which is convoluted, batch normalized, and finally activated by ReLU. F (∙) denotes combining the enhanced patches again into a complete feature map.γ and β are learnable parameters that control the contribution of the enhanced feature map to the original feature map ${f_{a},f}_{b}$, respectively. O is the signature map of the final output, which may be adjusted using adaptive average pooling if necessary when adjusted to the target size.

#### 2.2.5. Loss Function

Our model employs a multitask learning framework that optimizes both classification and segmentation tasks. The overall loss function is as follows:

(14) $$L_{total}=L_{cls}+L_{seg}$$

where $L_{cls}$ is the classification loss, using the cross-entropy loss function, and $L_{seg}$ is the segmentation loss, the formulas for the classification loss function and segmentation loss function are as follows:

(15) $$L_{cls}=-[ylog\hat{y}+\left(1-y\right)log\hat{y}]$$

(16) $$\begin{array}{cc} L_{seg}=-(\frac{1}{N\times H\times W}) & \\ & \times \sum_{n=1}^{N}\sum_{h=1}^{H}\sum_{w=1}^{W}[M(n,h,w)\times log(p(n,h,w))+(1-M(n,h,w))\times log(1\\ & -p(n,h,w))] \end{array}$$

where N is the batch size, H is the height of the segmented prediction map and the true mask, W is the width of the segmented prediction map and the true mask, and M(n,h,w) represents the true label of the Ground truth Mask at the nth sample, hth row, and wth column. This value is either 0 (representing the true class) or 1 (the counterfeit class). p(n,h,w) represents the probability that the model predicts that the pixel in the nth sample, hth row, and wth column belongs to category 1 (positive category, counterfeit category). The classification task focuses on image level forgery detection, while the segmentation task focuses on pixel level forgery region location. Both complement each other and improve the overall performance of the model.

## 3. Results

### 3.1. Experimental Settings

To fully evaluate the performance of the deep forgery detection network proposed in this paper, we conduct experiments on several mainstream public datasets, including FaceForensics++ (FF++) [23], CelebDF-v1, and CelebDF-v2 [20].

FF++ dataset contains 1000 original videos and 4000 fake videos generated by Deepfakes, Face2Face, FaceSwap, and NeuralTextures, respectively. In the experiment, we uniformly use its highly compressed version (c23). CelebDF-v1 dataset uses the popular deep forgery technology at that time to tamper with the original celebrity videos. Forgery means include facial replacement, expression transfer, etc., so that the dataset can fully cover different types and degrees of forgery. CelebDF-v2 dataset contains 590 original videos and 5639 high-quality fake videos, which are considered more challenging because of their more subtle falsification traces; in terms of model performance evaluation, we mainly use two widely recognized metrics: Area Under the Curve (AUC), which comprehensively measures the ability of the model to distinguish between true and false samples, the closer its value is to 1, the better the performance. Accuracy (ACC), which is the proportion of correctly classified samples in the total sample, intuitively reflects the overall classification accuracy of the model.

The implementation details are as follows:

For backbone network, we use two parallel Xception networks as feature extractors for RGB stream and SRM feature stream, respectively. The dimensions of the input images were uniformly adjusted to 299 × 299 pixels and normalized to the range [0, 1]. We employed common image enhancement techniques such as flipping, contrast adjustment, and blurring. Additionally, we increased the diversity of the forged regions through random cropping while ensuring alignment between the annotations and the images. Adam is selected as optimizer, and its initial learning rate is set to 1 × 10^−4^, and the weight attenuation coefficient is set to 1 × 10^−5^, and the batch size during training is set to 32. In terms of training strategy, we adopt cosine annealing learning rate scheduling mechanism, and the total training epochs vary according to different experimental stages (for example, some preliminary convergence analysis may be based on fewer rounds such as 30 epochs, while the complete performance evaluation may take longer training time such as 100 epochs, depending on each experimental scenario). In order to improve the robustness and generalization ability of the model, a variety of data enhancement techniques are applied in the training process, including random horizontal flip, random rotation at small angles, and random adjustment of image brightness and contrast. In addition, to prevent the model from overfitting, we also introduce an early stopping strategy, that is, when the performance of the model on the validation set does not show significant improvement for several consecutive epochs, we will terminate the training early. All models were implemented on the PyTorch1.12.1 framework and trained and tested on NVIDIA RTX 4080 GPU hardware.

### 3.2. Contrast Experiment

#### 3.2.1. Domain Performance

Table 1 shows the results of training and testing on the FF++ dataset. In the FF++ dataset, DF (DeepFakes), F2F (Face2Face), FS (FaceSwap), and NT (NeuralTextures) are abbreviations for four main Deepfake manipulation methods, with each method corresponding to distinct generation techniques and forgery targets. To ensure fairness in the experiments, we first evaluated the in-domain performance of each method when trained and tested on the same dataset. It should be noted that the metrics in Table 1 are sourced from the corresponding papers.

As can be seen from the data in Table 1, our method has an average AUC of 0.995 across the four categories, outperforming several comparison methods including Xception, Face X-Ray, and PCL+ l2G. In the category of Neural Textures, which is difficult to detect, our method also achieves AUC of 0.995, showing good detection ability.

#### 3.2.2. Generalization Performance Across Datasets

To assess the generalization ability of the model, we trained the model on FF++ dataset and tested it on CelebDF-v1 and CelebDF-v2 datasets. Table 2 shows the results of testing across datasets.

**Figure 5:**
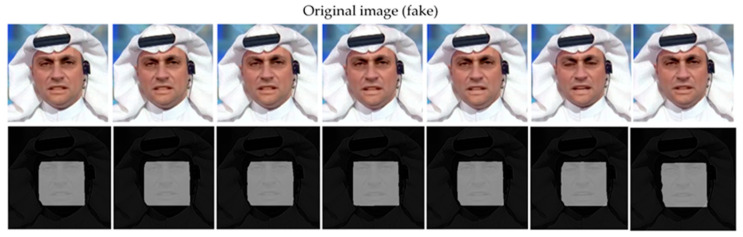
Effectiveness of the method described in this paper on actual dataset.

As can be seen from the data in Table 2, our method achieves the best performance in all cross-dataset tests, especially achieving an AUC value of 0.995 on the CelebDF-v2 dataset, which improves the performance by 9.1% compared to the sub-optimal method Locate and Verify [42]. This indicates that our proposed feature interaction and fusion mechanism significantly improves the generalization ability of the model across datasets. Figure 5 shows the effectiveness of our method on actual dataset. Our method can accurately segment the forged face regions.

### 3.3. Ablation Experiments

To validate the effectiveness of each module, we conducted a series of ablation experiments. Table 3 presents a performance comparison of different module combinations (trained and tested on the FF++ dataset).

As can be seen from Table 3, each of the proposed modules contributes to performance. In particular, the addition of the CFIE module raises the ACC from 0.901 to 0.934.

**Figure 6:**
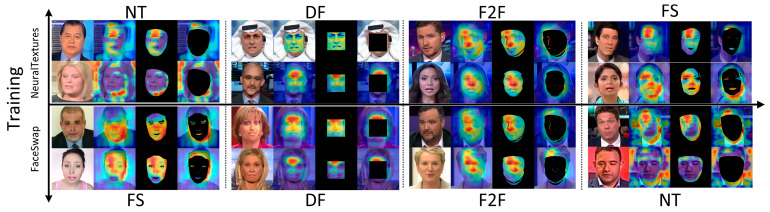
Response heat map visualization for different methods. The darker the color on the heatmap, the more attention the model pays to that area.

In order to understand our method more intuitively, we conducted feature visualization analysis. Figure 6 shows a heat map of the responses of different methods to counterfeit images under Grad-CAM mapping of the classification flow from our model.

As can be seen from Figure 6, our method can more accurately locate counterfeit areas, especially in facial details (such as eyes, mouth, etc.). Through the feature interaction of the CFIE module and attention enhancement of EFG module, the model can better capture forgery traces, which is manifested as a high response value of forgery area in the heat map.

**Figure 7:**
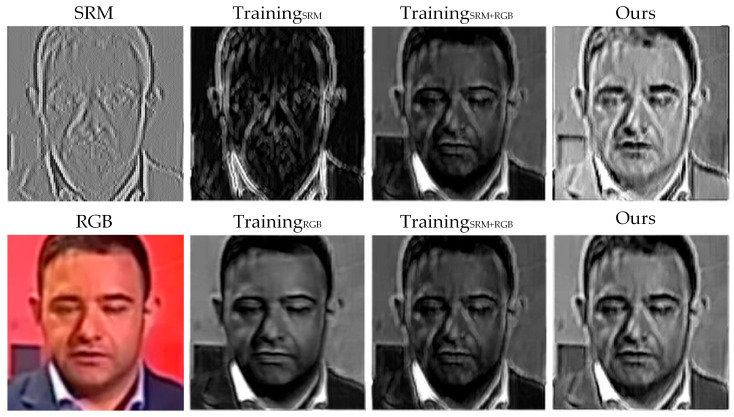
Visualization of CFIE module feature map.

In addition, we visualize the feature map in the CFIE module, as shown in Figure 7.

TrainingRGB and TrainingSRM represent features of an Xception model trained on a single modality. TrainingRGB +SRM is obtained by a two-stream model in which the two modes are summed directly. It can be seen from Figure 7 that the CFIE module effectively enhances the complementary features of RGB stream and SRM stream through cosine similarity guided feature interaction, so that the model can better capture forgery traces. In particular, the feature map processed by the CFIE module shows obvious activation response in forgery region, which proves the effectiveness of the CFIE module in forgery detection.

## 4. Discussion

The performance evaluation results presented in this study demonstrate that our proposed model, EFMID-Net, achieves outstanding AUC scores on the FF++ dataset. It not only significantly outperforms state-of-the-art methods such as DCL [33] and SBIs [35], but also achieves a remarkable AUC of 0.995 on the challenging NeuralTextures category. This result validates the effectiveness of the deep interaction mechanism between RGB and SRM features within our model design, which captures semantic consistency in original visual information while simultaneously reinforcing characteristic noise distribution patterns of forgery traces in SRM images, thereby enabling robust detection capabilities.

In cross-dataset evaluations, our model demonstrates strong performance, particularly achieving an AUC of 0.995 on the CelebDF-v2 dataset, representing a 9.1% improvement over the next best method, Locate and Verify [42]. Given that CelebDF-v2 employs more advanced generation techniques and exhibits significant distributional differences from FF++’s data sources, our feature interaction and fusion mechanism effectively overcomes challenges posed by data distribution shifts by learning cross-modal universal forgery patterns. This strong generalization capability indicates that the model maintains reliable detection performance when encountering unseen forgery techniques and scenarios in practical applications, thereby providing enhanced technical support for real-world deepfake defense. Although the current EFIMD-Net architecture significantly improves performance, its dual-stream design and multi-stage feature fusion mechanism introduce high computational complexity. Under the input size of 299 × 299, the baseline network Locate and Verify and EFIMD-Net are compared in terms of FLOPs and parameters (as shown in Table 4). Based on actual testing, we obtained a processing speed of approximately 9 fps (RTX4080 laptop).

## 5. Conclusions

To address the limitations of existing deepfake detection methods, including insufficient feature representation capabilities, low efficiency in multi-domain information fusion, and limited generalization across datasets, this paper proposes a deepfake detection network called EFIMD-Net, which is based on enhanced feature interaction and spatiotemporal domain fusion. By designing the CFIE module, EFG module, and EMFF module, our method can more effectively capture forgery traces and improve detection accuracy and generalization capabilities. The experimental results show that our method achieves AUC performance comparable to or even higher than the baseline Xception network on the FF++ dataset and its subsets. Additionally, compared to the two-stream network Locate and Verify, EFIMD-Net demonstrates superior performance on the CelebDF-v1 and CelebDF-v2 datasets, achieving 9% and 7% improvements in the AUC, respectively. The outstanding performance in cross-dataset testing demonstrates the effectiveness and generalization ability of the proposed method. Although the current EFIMD-Net architecture significantly improves performance, its dual-stream design and multi-level feature fusion mechanism introduce high computational complexity. In the future, we will explore lighter-weight models to adapt to resource-constrained deployment environments. The code is available at https://github.com/pwynb111/Enhanced-Feature-Interaction-and-Multi-Domain-Fusion-Deep-forgery-detection-network (accessed on 8 September 2025).

## Figures and Tables

**Table 1:** In-domain performance. The arrows indicate improvements in performance relative to the suboptimal results.

Method	FF++	DF	F2F	FS	NT	Avg
Xception [23]	0.963	0.994	0.995	0.994	0.995	0.942
Face X-Ray [32]	0.985	0.991	0.993	0.992	0.993	0.922
DCL [33]	0.993	-	0.992	-	0.990	0.991
PCL + l2G [34]	0.991	1.00	0.990	0.999	0.976	0.912
SBIs [35]	0.992	-	-	0.988	0.996	0.992
Ours	0.995 ↑0.002	0.997	0.997 ↑0.002	0.993	0.995	0.995 ↑0.003

**Table 2:** Comparison of generalization performance across datasets (AUC). The arrows indicate improvements in performance relative to the suboptimal results.

Method	Training Set	CelebDF-v1	CelebDF-v2
Xception [23]	FF++	0.623	0.737
Face X-Ray [32]	Prd	0.806	-
FWA [3]	Prd	0.538	0.569
DAM [36]	FF++	-	0.783
Li.et.al [37]	FF++	-	0.870
FTCN [38]	FF++	-	0.869
LiSiam [39]	FF++	0.811	0.782
SBIs [35]	Prd	-	0.870
LipForensics [40]	FF++	-	0.824
LITTD [41]	FF++	-	0.893
Locate and Verify [42]	FF++	0.847	0.922
Ours	FF++	0.938 ↑0.091	0.995 ↑0.073

**Table 3:** Ablation experiment results.

Model Variant	FF++	
	ACC	AUC
Xception	0.885	0.959
+ SRM	0.901	0.974
+ SRM + CFIE	0.934	0.989
+ SRM + CFIE + EFG	0.947	0.992
+ SRM + CFIE + EFG+EMFF	0.960	0.995

**Table 4:** Comparison of Locate and Verify with EFIMD-Net in FLOPs and parameters.

Method	FLOPs [G]	Parameters [M]
Locate and Verify [42]	21.39	61.87
EFIMD-Net (ours)	101.33	69.43

## Data Availability

The dataset used in this study is publicly available in multiple repositories on GitHub. It includes FF++, Celebdfv1 and Celebdfv2, featuring various deepfake images. [FF++, celebdfv1 and celebdfv2] [https://github.com/ondyari/FaceForensics (accessed on 8 September 2025). https://github.com/yuezunli/celeb-deepfakeforensics (accessed on 8 September 2025)].
